# Correction: Leadership development as a novel strategy to mitigate burnout among female physicians

**DOI:** 10.1371/journal.pone.0331323

**Published:** 2025-08-28

**Authors:** Dawn M. Sears, Alexis Bejcek, Laurel Kilpatrick, Nicole Griggs, Lindsey Farmer, Brittany Jackson, Hania Janek, Anthony C. Waddimba

The second author’s name is spelled incorrectly. The correct name is Alexis Bejcek. The correct citation is: Sears DM, Bejcek A, Kilpatrick L, Griggs N, Farmer L, Jackson B, et al. (2025) Leadership development as a novel strategy to mitigate burnout among female physicians. PLoS ONE 20(3): e0319895. https://doi.org/10.1371/journal.pone.0319895

There are errors in the author affiliations. The correct affiliations are as follows:

Dawn M. Sears^1^, Alexis Bejeck^2^, Laurel Kilpatrick^2^, Nicole Griggs^1^, Lindsey Farmer^3^, Brittany Jackson^4^, Hania Janek^5,6^, Anthony C. Waddimba^7,8,9^

1 North Texas VA Medical Center, Dallas University of Texas Southwestern Medical Center, Dallas, Texas, United States of America, 2 Department of Internal Medicine, Scott and White Medical Center, Baylor Scott and White Health, Temple, Texas, United States of America, 3 Department of Internal Medicine, UTHealth McGovern Medical School, Houston, Texas, United States of America, 4 Department of Anesthesiology, Vanderbilt University, Nashville, Tennessee, United States of America, 5 Office of Medical Education, Baylor Scott & White Health, 6 Department of Education, Innovation and Technology, Baylor College of Medicine, 7 Department of Surgery, Baylor University Medical Center, Baylor Scott and White Health, Dallas, Texas, United States of America, 8 Baylor Scott and White Research Institute, Baylor Scott and White Health, Dallas, Texas, United States of America, 9 Department of Medical Education, Texas A&M College of Medicine, Dallas, Texas, United States of America

## References

[1] Sears DM, Bejeck A, Kilpatrick L, Griggs N, Farmer L, Jackson B, et al. Leadership development as a novel strategy to mitigate burnout among female physicians. PLoS One. 2025;20(3):e0319895. doi: 10.1371/journal.pone.0319895 40100847 PMC11918409

