# Supplementary material for: Leadership development as a novel strategy to mitigate burnout among female physicians
Source: PLoS One. 2025 Mar 18;20(3):e0319895. doi: 10.1371/journal.pone.0319895 (PMC11918409; doi:10.1371/journal.pone.0319895)
Supplement: S1 Table — (PDF) [file pone.0319895.s001.pdf]

S1 Table: Nonparmetric Multivaiate Repeated Measures Analysis of Variance By Ranks on the Outcome Scores

Comparison Group		**Intervention Group** BSWH Female Physicians Who Attended WLiM Events			**Female Comparison Group** BSWH Female Physicians Who Did Not Attend WLiM Events (cohort 1)			**Male Comparison Group** BSWH Male Physician Respondents (Cohort 2)		
Outcome Variable	Measure / Scale	Difference in average ranks ($T_2 – T_1$)	95% CI	Dunnett's Multiple Comparisons T-test	Difference in average ranks ($T_2 – T_1$)	95% CI	Dunnett's Multiple Comparisons T-test	Difference in average ranks ($T_2 – T_1$)	95% CI	Dunnett's Multiple Comparisons T-test
Emotional Exhaustion (EE)	3-Item Abbreviated Subscale	-0.0765	-0.459 - 0.306	p>0.05	0.204	-0.196 - 0.605	p>0.05	-0.0107	-0.220 - 0.198	p>0.05
	"I feel burned out"	**-0.271**	**-0.539 - -0.00360**	**p<0.05**	-0.0833	-0.312 - 0.146	p>0.05	-0.102	-0.253 - 0.0489	p>0.05
Depersonal-ization (DP)	3-Item Abbreviated Subscale	**-0.425**	**-0.736 - -0.115**	**p<0.05**	-0.136	-0.453 - 0.181	p>0.05	0.0206	-0.167 - 0.208	p>0.05
	"more callous"	**-0.253**	**-0.483 - -0.0234**	**p<0.05**	0.0167	-0.225 - 0.258	p>0.05	-0.095	-0.236 - 0.046	p>0.05
Personal Accomplish-ment (PA)	3-Item Abbreviated Subscale	0.0114	-0.348 - 0.371	p>0.05	0.242	-0.167 - 0.651	p>0.05	0.061	-0.143 - 0.265	p>0.05
	"accompli-shed"	-0.126	-0.411 - 0.160	p>0.05	-0.0214	-0.243 - 0.200	p>0.05	-0.108	-0.243 - 0.0280	p>0.05
Leadership Aspiration	"to what extent do you aspire to be a leader"	0.111	-0.155 - 0.378	p>0.05	**-0.331**	**-0.598 - -0.0628**	**p<0.05**	-0.0133	-0.146 - 0.120	p>0.05
Intention to										

Stay (Retention)	"plan on staying with organization for the next 5 years"	-0.163	-0.389 - 0.0633	p>0.05	0.011	-0.228 - 0.251	p>0.05	-0.0135	-0.140 - 0.113	p>0.05
