# Supplementary material for: Leadership development as a novel strategy to mitigate burnout among female physicians
Source: PLoS One. 2025 Mar 18;20(3):e0319895. doi: 10.1371/journal.pone.0319895 (PMC11918409; doi:10.1371/journal.pone.0319895)
Supplement: S2 Table — (PDF) [file pone.0319895.s002.pdf]

S2 Table: Friedman's Unadjusted Repeated Measures Nonparametric Analyses of Variance By Rank on the Outcome Scores.

Comparison Group		Intervention Group<br>BSWH Female Physicians Who Attended WLiM Events			Female Comparison Group<br>BSWH Female Physicians Who Did Not Attend WLiM Events (Cohort 1)			Male Comparison Group<br>BSWH Male Physician Respondents (Cohort 2)		
Outcome Variable	Measure / Scale	Difference in average ranks ($T_2 - T_1$)	95% CI	Dunnett's Multiple Comparisons T-test	Difference in average ranks ($T_2 - T_1$)	95% CI	Dunnett's Multiple Comparisons T-test	Difference in average ranks ($T_2 - T_1$)	95% CI	Dunnett's Multiple Comparisons T-test
Emotional Exhaustion (EE)	3-Item Abbreviated Subscale	-0.179	-0.413 - 0.055	p>0.05	**+0.131**	**0.054 - 0.209**	**p<0.05**	**+0.088**	**0.039 - 0.138**	**p<0.05**
	"I feel burned out"	**-0.291**	**-0.472 - -0.111**	**p<0.05**	-0.027	-0.210 - 0.156	p>0.05	-0.071	-0.191 - 0.050	p>0.05
Depersonali-zation (DP)	3-Item Abbreviated Subscale	**-0.432**	**-0.674 - -0.190**	**p<0.05**	**+0.089**	**0.012 - 0.166**	**p<0.05**	**+0.089**	**0.045 - 0.134**	**p<0.05**
	"more callous"	**-0.194**	**-0.379 - -0.010**	**p<0.05**	+0.052	-0.116 - 0.220	p>0.05	-0.049	-0.162 - 0.064	p>0.05
Personal Accomplish-ment (PA)	3-Item Abbreviated Subscale	-0.0308	-0.285 - 0.223	p>0.05	**+0.103**	**0.033 - 0.173**	**p<0.05**	**+0.136**	**0.082 - 0.190**	**p<0.05**
	"accompli-shed"	-0.145	-0.312 - 0.022	p>0.05	0.106	-0.024 - 0.236	p>0.05	-0.083	-0.176 - 0.011	p>0.05
Leadership Aspiration	"to what extent do you aspire to be a leader"	0.040	-0.157 - 0.236	p>0.05	**-0.205**	**-0.376 - -0.034**	**p<0.05**	-0.00737	-0.116 - 0.101	p>0.05

Intention to Stay (Retention)	"plan on staying with organization for the next 5 years"	**-0.184**	**-0.349 - -0.019**	**p<0.05**	+0.00994	-0.149 - 0.169	p>0.05	-0.018	-0.117 - 0.082	p>0.05
